# Pudding Proteomics: Cyclomaltodextrin Glucanotransferase and Microbial Proteases Can Liquefy Extended Shelf Life Dairy Products

**DOI:** 10.3390/metabo12030254

**Published:** 2022-03-17

**Authors:** Kristina J. H. Kleinwort, Maria Weigand, Lydia Hoffmann, Roxane L. Degroote, Richard Dietrich, Erwin Märtlbauer, Stefanie M. Hauck, Cornelia A. Deeg

**Affiliations:** 1 Department of Veterinary Sciences, LMU Munich, D-82152 Martinsried, Germany; k.kleinwort@lmu.de (K.J.H.K.); maria.weigand@lgl.bayern.de (M.W.); lydia.hoffmann@tiph.vetmed.uni-muenchen.de (L.H.); r.degroote@lmu.de (R.L.D.); 2 Department of Veterinary Sciences, LMU Munich, D-85764 Oberschleißheim, Germany; r.dietrich@mh.vetmed.uni-muenchen.de (R.D.); e.maertlbauer@mh.vetmed.uni-muenchen.de (E.M.); 3 Metabolomics and Proteomics Core, Helmholtz Center Munich, German Research Center for Environmental Health, D-80939 Munich, Germany; hauck@helmholtz-muenchen.de

**Keywords:** pudding, liquefaction, α-amylase, CGTase, *Pseudomonas*, proteomics, zymography, quantitative label-free LC-MS/MS

## Abstract

In recent years, a lack of stability of dairy products with extended shelf life (e.g., yoghurt products, UHT desserts) has occurred, with the corresponding products liquefying significantly after days or weeks. This project aimed to identify the enzymes responsible for the liquefaction of the affected products based on differential proteomic analyses. No evidence was found for the presence of starch-degrading bacteria in the affected products. With zymography and proteome analysis, we detected the cause of liquefaction in a pudding by contamination of its aroma component with an engineered amylolytic enzyme, cyclomaltodextrin glucanotransferase (CGTase) from *Thermoanaerobacterium thermosulfurigenes*. In addition, we detected contamination with *Pseudomonas*-derived proteolytic ATP-dependent Clp protease in one pudding batch and proteases in technically used amylases, which degraded β-caseins in another batch. Identification of these agents with liquefying properties in dairy products are useful for adjustment of production protocols and/or composition of additives, and thus shelf life extension.

## 1. Introduction

In recent years, the growing demand for non-refrigerated dairy products with extended shelf life has led to increased production of starchy and heat-treated dairy products (e.g., yoghurt products, ultra-high temperature (UHT) desserts) [1]. However, there have been increasing problems with the stability of these novel dairy products [2], leading to almost complete liquefaction. The products liquefy in individual batches and are characterized by almost complete degradation of the modified starch added as a thickener. As the changes often take weeks to appear in the affected batches, customer complaints and claims for compensation are made. It is important to understand the underlying cause of liquefaction to identify the affected products before shipment or even to prevent the production of such food products.

Although proteases might be associated with this problem in some cases, liquefaction is mainly attributed to the activity of starch-degrading enzymes, especially α-amylases [3].

However, it is still unclear whether these enzymes were first formed in the final product or whether the final product was contaminated by crude products. In principle, starch degradation can be caused by both de novo synthesized or preformed amylases. Therefore, contamination with microorganisms producing degradative enzymes should also be investigated. Bacteria produce the greatest diversity of α-amylases; heat-stable variants are produced in particular by the food-relevant species *B. amyloliquefaciens*, *B. licheniformis*, and *G. stearothermophilus*. Since the affected products are subjected to a heating process, usually UHT treatment, the causative microorganisms or enzymes must exhibit certain temperature stability.

Such properties are typical for spore formers and their enzymes, which are used in the production of sugar syrups, but also as ingredients of detergents. Moreover, industrial starch liquefaction and saccharification are carried out by enzymatic hydrolysis using various amylases (mainly α-amylases, but also ß- and γ-amylases), most of which are derived from *Bacillus* spp. [4,5]. However, thermostable or thermotolerant α-amylases with effectiveness in a variety of pH ranges are also produced by many other microorganisms, such as *Geobacillus*, *Micrococcus*, and *Alicyclobacillus* species [4,6].

This means that amylases can originate from many microorganisms and can come either from contamination with undesirable pathogens or from technical or dairy sources and can lead to starch degradation in different ways. Therefore, this study aimed to identify the enzyme(s) and source responsible for the liquefaction of dairy products by differential proteomic analyses.

## 2. Results

### 2.1. Identification and Further Characterization of Added Amylases in Yoghurt 

Differential proteomic analyses allow the detection of unknown proteins from different species. However, there are certain samples where identification is hindered by the unfavorable dynamic range of the proteins in the sample, e.g., in milk or milk products that have a similar composition to serum [7], for which the dynamic range of the concentration is at least 10 orders of magnitude [8].

Casein accounts for 80% of the total protein content in dairy products, making it difficult to detect smaller amounts of other proteins [9]. Various protocols for milk protein extraction have been described in the literature, and the diversity of methods indicate that there is no established method that is superior to the others for complete proteomic analysis in milk [9].

**Figure 1:**
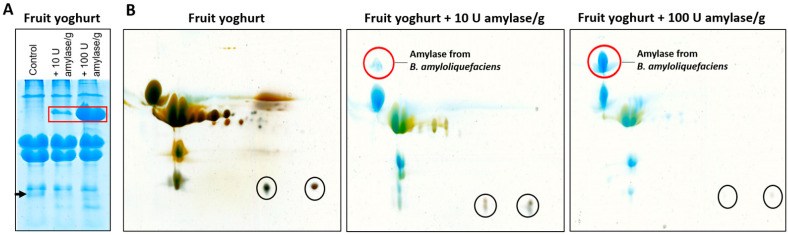
Fruit yoghurt spiked with amylase. (**A**) 1D SDS gel, colloidal Coomassie staining, and (**B**) 2D SDS gels of control fruit yoghurt and fruit yoghurt spiked with different amounts of amylase from *B. amyloliquefaciens* (colloidal Coomassie staining followed by silver staining). The red markers indicate the amylase bands or stains. The arrow and black circles indicate proteins with a molecular weight of approximately 18 kDa that were digested by amylase, as they weakened or disappeared with increasing amylase concentration.

For dairy products such as yoghurt or pudding, analyses are further complicated by sample preparation from this complex matrix. Therefore, we first established a protocol for processing yoghurt for proteomic analyses in 1D and 2D gels to separate the complex proteome. In addition, different amounts of commercially available purified amylases were added to the yoghurts at different concentrations to determine the amount that could be visualized on the gel. The major proteins of fruit yoghurt were clearly visible in the gels, confirming the predominance of a few proteins making up 95% of the yoghurt proteome, while the many proteins with lower abundance were not detectable with colloidal Coomassie or silver staining (Figure 1). Spiked amylase from *B. amyloliquefaciens* was visible from 10 U per g yoghurt. In the gels containing fruit yoghurt and amylase, an interesting side finding occurred. In the 1D SDS gel, a yoghurt protein with a molecular weight of approximately 18 kDa was increasingly digested with rising amounts of added amylase (Figure 1A, arrow). The 2D SDS gels showed that these were two proteins with the same molecular weight but slightly different isoelectric points (Figure 1B, black circles). To identify these proteins, the protein bands and spots were cut from the gels, digested, and analyzed by mass spectrometry. The protein identified in all three samples was β-casein (Table S1).

### 2.2. β-Casein Cleavage by Amylase Preparation from B. amyloliquefaciens

**Figure 2:**
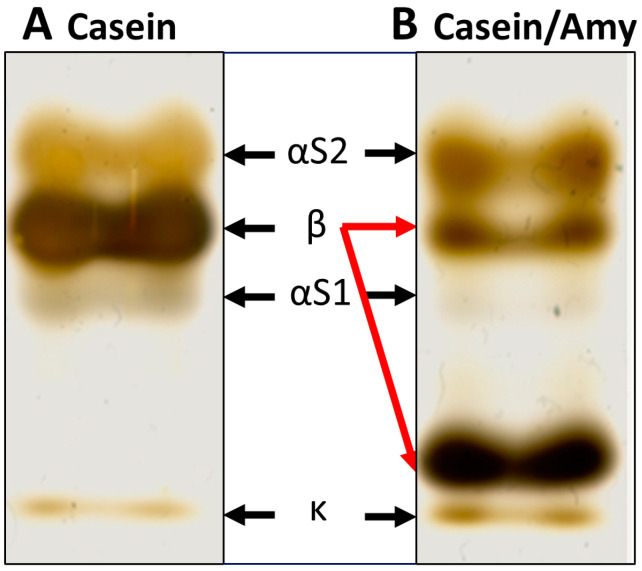
Casein digestion by amylase preparation (silver staining). (**A**) 1D SDS gel of purified casein from cow’s milk. (**B**) After incubation with amylase preparation from *B. amyloliquefaciens*, mainly the β-casein band, but also the αS1-casein band appear weaker. In addition, another band appears directly above the k-casein band (red arrows).

To confirm this finding of casein degradation after incubation with the amylase preparation from *B. amyloliquefaciens*, purified casein from cow’s milk was pre-incubated with this amylase (0.625 U/mg) and then separated in a 1D SDS gel (Figure 2).

Of the casein proteins (Figure 2A), mainly β-casein, but also αS1-casein, were digested and degraded into smaller fragments by the added amylase preparation (Figure 2B). With this experiment, we confirmed that the proteins digested by the commercial amylases were indeed caseins and primarily β-casein.

Casein digestion was observed for two different commercially available amylase preparations from *A. oryzae* and *B. amyloliquefaciens*. To determine whether these carbohydrate-specific amylases can also digest proteins as a moonshine activity or whether the respective preparations contained proteases, we analyzed the composition of these two purified amylases from *Aspergillus oryzae* (Sigma-Aldrich A8220) and *Bacillus amyloliquefaciens* (Sigma-Aldrich A7595), as well as from *Bacillus licheniformis* (Sigma-Aldrich A4862) by mass spectrometry. A protease from *Achromobacter lyticus* was detected in the amylase preparations from *A. oryzae* and *B. amyloliquefaciens*, but not in the purified amylase from *B. licheniformis* (Table S2). Therefore, the observed casein degradation was a contamination with proteases in the two purified amylase preparations from *A. oryzae* and *B. amyloliquefaciens*.

### 2.3. Identification of Cyclomaltodextrin Glucanotransferase from the Aroma of Liquefied Pudding

To determine the cause of the pudding decay, retention samples of liquefied chocolate pudding batches and a control pudding from the same manufacturer, as well as the aroma of the liquefied pudding, were analyzed. SDS gel electrophoresis did not detect any bands or stains pointing to an amylase in the affected pudding or the aroma used for flavoring (data not shown).

**Figure 3:**
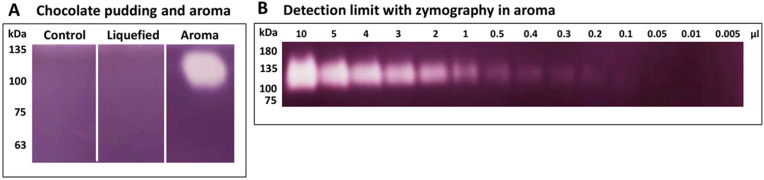
Assay for starch degrading enzymes by zymography. (**A**) No activity detectable in control pudding or liquefied pudding, but in aroma from liquefied pudding (right trace, white spot indicates digestion of starch). (**B**) Starch digestion in respective aroma is detectable down to 0.1 µL. The detection limit of this assay was investigated to be around 0.5–20 ng, depending on the present amylase.

Since the presence and activity of enzymes can also be detected by zymography, a method in which the approximate molecular weight of the enzyme is determined by separation in the gel and enzyme activity is visualized by conversion of the substrate [10], we used this technique to detect the activity of starch-degrading enzymes in the liquefied samples and their components (Figure 3).

**Figure 4:**
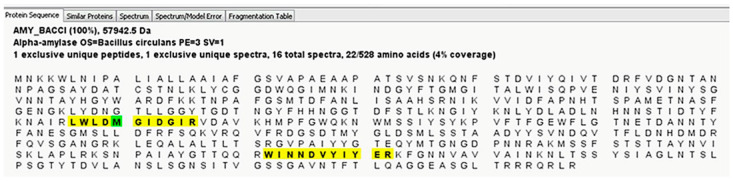
Identification of alpha-amylase in aroma from liquefied pudding. Sequence coverage of alpha-amylase from *Bacillus circulans* (detected peptides are highlighted in yellow, amino acids with post-translational modifications are shown in green) from a representative mass spectrometry run. Sequence coverage of AMY_BACCI was 4%.

To identify this starch-degrading enzyme, we analyzed the respective aroma and the pudding samples by mass spectrometry. Again, differential proteomic analysis of control and liquefied pudding revealed no starch-degrading enzyme. However, in the aroma, an α-amylase from *Bacillus circulans* (Figure 4, Table S3) was detected.

**Figure 5:**
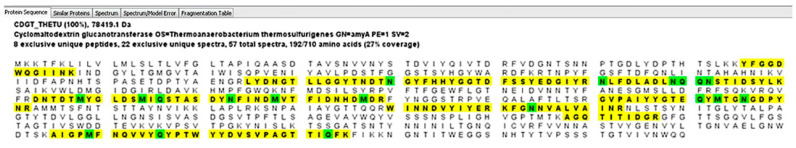
Identification of a CGTase in aroma from the liquefied pudding. Yellow-labeled sequences, covering 27% of the CDGT_THETU sequence, were identified.

Interestingly, a cyclomaltodextrin glucanotransferase (CGTase) from *Thermoanaerobacterium thermosulfurigenes* was also detected, with a sequence coverage of 27% (Figure 5, Table S3).

CGTases are also starch-degrading enzymes and belong to the group 13 family of glycoside hydrolases, which consists of more than 80 enzymes [11,12]. CGTases are used industrially to modify starch [13]. They convert starch into linear and cyclic oligosaccharides, so-called ring-shaped dextrins (cyclodextrins) [14].

### 2.4. Detection of Protease from Pseudomonas Contamination in Liquefied Pudding

**Figure 6:**
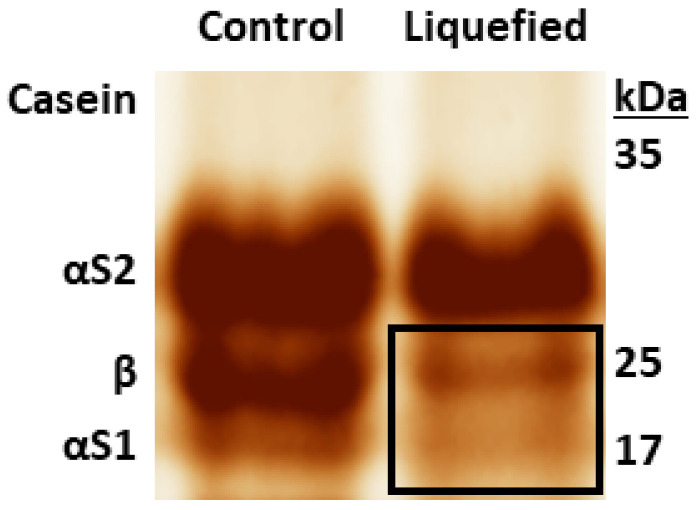
Control pudding and liquefied vanilla pudding in SDS gel (silver staining). In the liquefied sample, a much weaker band of β-casein, but also of αS1-casein was detected (right lane, black inset).

A liquefied vanilla pudding from respective batch of liquefied products with an associated non-liquefied control batch was also analyzed for amylases or starch-degrading enzymes. However, no starch degradation was detected in the liquefied pudding. Instead, casein degradation was detectable in the liquefied sample (Figure 6).

Differential proteomic analyses revealed that no starch-degrading enzymes were present in the pudding, but the pudding from the liquefied batch contained 81 proteins of *Pseudomonas* spp. (Table S4), indicating microbial contamination in this case. Among the corresponding proteins, the *Pseudomonas* protease “proteolytic ATP-dependent Clp protease” was detected in the degraded vanilla pudding (Table S4).

## 3. Discussion

The sweet taste is subconsciously linked to safety and happiness [15] and milk-based desserts are a popular food for many consumers [16]. They are simple to prepare and usually consist of milk (skim or whole), thickener (usually starch or hydrocolloids), colorant, aroma, and sugar or sugar substitutes [16]. Alterations of the original recipes are created to meet consumer’s needs, e.g., products with extended shelf life for export. Significant changes in rheological properties may occur in these products; in particular, changes in texture and even liquefaction can occur. It was the goal of this project to identify the cause of liquefaction in dairy products. We were able to find several of these causes in the retained samples of respective affected charges examined in our study.

In model experiments, where we co-incubated fruit yoghurts with purified amylases that are used as industrial enzymes to hydrolyze starch for food products, we detected a marked β-casein degradation by some of the purified α-amylase preparations (Figure 1 and Figure 2, Table S1). To test if these amylases were moonlighting proteins or if an unrecognized contaminant caused the proteolytic activity of the commercial enzyme preparations, we analyzed their composition with mass spectrometry. Moonlighting proteins are members of protein superfamilies that share significant amino acid sequences and structural homology, yet catalyze different reactions or act on different substrates [17]. In moonlighting enzymes, the protein has a catalytic activity and a second, unrelated activity [18,19,20]. Over 300 moonlighting proteins have been identified so far [21].

Among α-amylases, the most intensively studied enzymes degrading starch substrates widely used in various branches of the food, pharmaceutical, and chemical industries, mainly belong to family 13 of the glycoside hydrolases (GH13) [22,23,24]. Multifunctional amylases, such as maltogenic amylases, form a special subfamily within the GH13 family because they have some unique catalytic properties compared to other enzymes in the α-amylase family [25,26]. Multifunctional amylases possess transglycosylation and hydrolysis activity for various glucan substrates, leading to the production of isomaltooligosaccharides and maltooligosaccharides as well as glucose [27]. Thermophilic multifunctional amylase OPMA-N has strong α-1,6-transglycosylation activity and α-1,4-hydrolytic activity on starch [27]. However, mass spectrometric analysis of the purified amylase preparations of *A. oryzae* and *B. amyloliquefaciens* that exhibited casein degradation in our studies, especially of β-casein, revealed that both contained proteases, in contrast to the amylase of *B. licheniformis* that did not degrade caseins (Table S2). Therefore, we attributed the caseinolytic activity to the protease of *Achromobacter lyticus* identified by mass spectrometry. Being able to break down casein into peptides and amino acids, this serine protease is alternatively named caseinase [28]. It is used to produce bacterial enzymes [29] and therefore most likely originates from production process of recombinant amylases.

In order to detect the cause of liquefaction in a batch of chocolate pudding, we analyzed the liquefied pudding, a respective unliquefied control pudding as well as the used chocolate pudding aroma with zymography and mass spectrometry. We could not detect any evidence of starch-degrading enzymes in the degraded pudding and the unliquefied control. However, in the chocolate pudding aroma, a starch-degradation was seen with zymography (Figure 3). With mass spectrometry, we then identified a cyclomaltodextrin glucanotransferase (CGTase) from *Thermoanaerobacterium thermosulfurigenes* in the aroma (Figure 5, Table S3). CGTases are used in the starch industry to modify starch [30,31,32,33,34]. They convert starch into linear and cyclic oligosaccharides, so-called ring-shaped dextrins (cyclodextrins) [30,31,32,33]. These cyclodextrins are used in food additives as carriers of aromas, to stabilize fat-soluble colorants and to improve aroma (e.g., to reduce the undesirable aftertaste in sweeteners) [12,35]. CGTases are optimized for industrial use through targeted mutations [14,36]. For example, in CGTase from *P. macerans*, a mutation at position 47 from lysine to threonine (or leucine or serine) results in decreased α-cyclodextrin formation activity and increased β-cyclodextrin formation [14]. Very thermostable CGTases are generated by genetic mutations, but they can also be found in specialized bacteria and archae [12,35,37]. For the CGTase from *T. thermosulfurigenes* detected here, the temperature optimum is >60–100 °C (as listed in BRENDA. Available online: https://www.brenda-enzymes.org (accessed on 12 February 2022)). Extremophile-derived α-amylases are tolerant of extreme pH, temperature, and various forms of environmental stress [3,38]. Therefore, the respective enzyme might still be active after UHT treatment. Amylases are widely used in the food processing industry, e.g., in baking, brewing, preparation of digestive aids, and the production of cakes, fruit juices, and starch syrups [39]. α-amylases from *B. licheniformis* and *B. amyloliquefaciens* are known for their enormous heat resistance and are therefore used for liquefaction and saccharification of starch in the sugar industry, as well as naturally occurring and genetically modified CGTases [6,12,35,37]. There is a genetically modified, patented CGTase that contains the same sequence (LWLDMGIDGIR) identified as peptide from *Bacillus circulans* amylase in our study. It is therefore quite possible that this genetically modified CGTase enzyme was present in the sample, but could not be identified due to the lack of an entry in the database. However, based on the experiments carried out, we also cannot rule out that the identified amylase was additionally present in the sample.

The CGTase detected here most likely came from the production of the pudding aroma and not from a contamination with *T. thermosulfurigenes*, because the performed tests for microbial contamination were negative in all of the products of this proteomic analysis (data not shown).

In contrast, in the differential proteome analyses of the liquefied vanilla pudding, we did not find proteins with starch degrading function, but several proteins of *Pseudomonas*, that were only present in the degraded product, but not in the control (Table S4). Among them was the “proteolytic ATP-dependent Clp protease” from *Pseudomonas savastanoi pv. phaseolicola*, which was detected exclusively in the liquefied pudding. Proteolytic ATP-dependent Clp protease is a stress-induced, ATP-dependent protease, that cleaves caseins (Clp = Caseinolytic peptidase) [40,41]. Therefore, we assume the respective protease from *Pseudomonas* contamination was the cause for liquefaction of the vanilla pudding. Thus, this proteomic finding explains the casein digestion detected in the experiments (Figure 6) and the degradation in the product batch.

## 4. Materials and Methods

### 4.1. Dairy Products, Casein, and Amylase Preparations

To establish a protocol for processing of dairy products for proteomic analyses in SDS gels, a randomly selected fruit yoghurt (strawberry yoghurt from cow’s milk) from the supermarket cooling shelf was used. Retention samples of liquefied pudding batches were provided directly by the manufacturers. The liquefied chocolate pudding, the respective aroma, and the associated unliquefied control pudding originated from a different manufacturer than the liquefied vanilla pudding and respective unliquefied control pudding. All dairy products and the aroma were kept in their original packaging at 4 °C until further use.

For verification of casein digestion through amylase preparations, purified casein from cow’s milk (Sigma-Aldrich, Taufkirchen, Germany) was used.

Three different amylase preparations were analyzed in this study, *Aspergillus oryzae* (A8220), *Bacillus amyloliquefaciens* (A7595), and *Bacillus licheniformis* (A4862, all from Sigma-Aldrich). To determine the amount of amylase in dairy products that can be visualized on a SDS gel, different amounts (10 U and 100 U/g yoghurt) of the amylase preparations were added to fruit yoghurt, incubated at 37 °C for 30 min and subsequently processed in 1D or 2D SDS Gels. For analysis of casein digestion through amylase preparations, the amylases were added to a casein solution (1 mg/mL) in a concentration of 0.625 U/mg casein and incubated for one day at 37 °C in the dark. Samples were then separated in 1D SDS gels.

### 4.2. 1D and 2D SDS-PAGE

To separate the fruit yoghurts in SDS gels, they were first processed as described [42]. Briefly, fruit yoghurt samples were diluted 1:2 with 35 mM NaCl and filtered through a coarse-pored filter to eliminate rough particles (e.g., fruit pieces). Finally, samples were acidified with 37% HCl to pH 2 and protein content of samples was determined by Bradford protein assay [43].

For 1D SDS-polyacrylamide gel electrophoresis (PAGE), the processed yoghurt samples were subsequently diluted 1:2 in lysis buffer (1% NP-40, 150 mM NaCl, 1× Roche Complete, 5 mM 2-Iodacetamide, 10 mM Tris-HCl, pH 7.6) and proteins were then separated in 12% SDS polyacrylamide gels (5 μg protein/slot) under denaturing and reducing conditions, followed by staining with colloidal Coomassie. 

For 2D SDS-PAGE, the processed yoghurt samples were lysed as described above, loaded onto 11 cm pH 3–11 NL IPG strips (GE Healthcare, Freiburg, Germany) by overnight reswelling (75 µg protein/strip) and subjected to isoelectric focusing (IEF) on a 2117 Multiphor II Electrophoresis Unit with an Amersham Electrophoresis Power Supply EPS 3501 XL (Step 1: 2 h/150 V/2 mA/5 W, Step2: 3 h/300 V/2 mA/2 W, Step 3: 13 h/1000 V/2 mA/5 W). Strips were then equilibrated in 1% Dithiothreitol followed by 4.8% Iodoacetamide for 10 min each and applied on 12% SDS polyacrylamide gels where proteins were separated. Resulting gels were stained with colloidal Coomassie followed by silver staining. For silver staining, Coomassie stained 2D SDS gels were washed three times for rehydration and subsequently incubated twice for 20 min in a solution containing 50% methanol, 12% acetic acid and 0.0185% formaldehyde in Aqua bidest. After three incubations for each 10 min in 50% ethanol, gels were soaked in 0.8 mM Na_2_S_2_O_3_ for 15 s. Next, they were shortly washed twice with Aqua bidest and then incubated for 20 min in a 11.8 mM AgNO_3_ solution containing 0.028% formaldehyde. Gels were again washed in Aqua bidest, followed by incubation in staining solution (0.57 M Na_2_CO_3_, 0.02 mM Na_2_S_2_O_3_, 0.0185% formaldehyde) until staining of light to dark brown protein spots became visible. The staining was stopped through incubation of gels in 50% methanol and 12% acetic acid for at least 10 min. Finally, the gels were incubated in 20% ethanol and 2% glycerin for at least 20 min. 1D and 2D SDS gels were scanned with an Amersham ImageQuant 800 biomolecular imager (GE Healthcare).

### 4.3. Zymography

Starch-degrading activity in dairy products or of purified enzymes was determined with starch zymography. Chocolate pudding aroma as well as liquefied and control chocolate pudding were diluted 1:2 with 35 mM NaCl for homogenization. An amount of 20 µg protein per pudding preparation as well as 0.005–20 µL of the aroma preparation were subjected to 1D SDS-PAGE under denaturing, but non-reducing conditions on 12% gels at 4 °C. SDS gels were incubated overnight in a 0.2 M phosphate buffer (pH 6) containing 1% starch at 4 °C, followed by 2 h incubation at 45 °C with fresh phosphate-starch buffer. Gels were washed in Aqua bidest and subsequently incubated in Lugol’s solution (Sigma-Aldrich) at room temperature until light bands became visible. The stained gels were washed again in Aqua bidest. Starch-degrading activity was detected as transparent bands on a dark background of undegraded substrate. Gels were scanned with an Amersham ImageQuant 800 biomolecular imager (GE Healthcare).

### 4.4. Sample Digestion for Differential Proteome Analysis

For identification of the yoghurt proteins digested from amylase preparations, selected band from 1D and spots from 2D gels were manually excised, washed with water, and destained by repetitive washes in water and in buffer containing 30 mM potassium ferricyanide and 100 mM sodium thiosulfate. After washing with water, gel pieces were shrunk in 100% acetonitrile (ACN) and rehydrated in ammonium bicarbonate (ABC; 50 mM NH_3_HCO_3_ diluted in HPLC-grade water) buffer. This treatment was repeated once, followed by discarding the supernatant and drying the gel pieces in a SpeedVac centrifuge. For digestion, spots were overlayed with 0.01 mg/mL trypsin in 50 mM ABC buffer and incubated overnight at 37 °C. The supernatant was collected and combined with the eluates of subsequent elution steps with 80% ACN and 0.1% trifluoroacetic acid (TFA). The combined eluates were dried in a SpeedVac centrifuge and dissolved in 2% ACN and 0.5% TFA for mass spectrometric analysis.

For mass spectrometric analysis of the amylase preparations and of the liquefied and the control puddings as well as the chocolate pudding aroma, samples were digested by a modified FASP protocol as described [44,45]. Briefly, samples were diluted 1:10 with 0.1 M tris/HCl (pH 8.5), and 100 mM dithiothreitol was added for 30 min at 60 °C. After cooling down, a UA buffer (8 M urea and 1 M tris-HCl, pH 8.5, diluted in HPLC-grade water) and 300 mM iodoacetamide were added and incubated for 30 min at room temperature in the dark. Eluates were transferred to 30 kDa cut-off centrifuge filters (Sartorius, Göttingen, Germany) and washed five times with UA-buffer and two times with ABC buffer. After washing, proteins were subjected to proteolysis for 2 h at room temperature with 0.5 µg Lys C in ABC-buffer, followed by the addition of 1 µg trypsin and incubation at 37 °C overnight. Peptides were collected by centrifugation and acidified with 0.5% TFA.

### 4.5. Mass Spectrometric Analysis and Protein Identification

Analysis of peptides was performed as described [46]. Acidified eluted peptides were analyzed in the data-dependent mode on a Q Exactive HF mass spectrometer (Thermo Fisher Scientific, Bremen, Germany) online coupled to an UItimate 3000 RSLC nano-HPLC (Dionex, Sunnyvale, CA, USA). Samples were automatically injected and loaded onto the C18 trap column, eluted after 5 min, and separated on the C18 analytical column (Acquity UPLC M-Class HSS T3 column, 1.8 µm, 75 µm × 250 mm; Waters) by a 90 min non-linear acetonitrile gradient at a flow rate of 250 nL/min. MS spectra were recorded at a resolution of 60,000, and after each MS1 cycle, the 10 most abundant peptide ions were selected for fragmentation. All MS/MS samples were analyzed using Mascot (Matrix Science, London, UK; version 2.6.2). Mascot was set up to search the SwissProt database (release 2020_04, 563,082 entries) or the Ensembl bovine database (release 75, 22,118 entries) assuming the digestion enzyme trypsin and allowing up to one missed cleavage site. Mascot was searched with a fragment ion mass tolerance of 0.020 Da and a parent ion tolerance of 10 ppm. Carbamidomethyl of cysteine was specified as a fixed modification. Deamidated of asparagine and glutamine and oxidation of methionine were specified as variable modifications.

Scaffold (version Scaffold_4.8.2, Proteome Software Inc., Portland, OR, USA) was used to validate MS/MS based peptide and protein identifications. Protein identifications were accepted if they could be established at greater than 95.0% probability and contained at least one identified peptide. Protein probabilities were assigned by the Protein Prophet algorithm (Nesvizhskii, Al et al. Anal. Chem. 2003; 75(17): 4646-58). Proteins that contained similar peptides and could not be differentiated based on MS/MS analysis alone were grouped to satisfy the principles of parsimony. Proteins sharing significant peptide evidence were grouped into clusters.

## 5. Conclusions

Zymography and proteome analyses with mass spectrometry enabled detection of enzymes that degraded texture of dairy products. Proteomics proved valuable for the detection of unknown interfering factors. We identified an industrially used starch-degrading enzyme, cyclomaltodextrin glucanotransferase from *Thermoanaerobacterium thermosulfurigenes*, as a cause for liquefaction of one of the analyzed batches of chocolate pudding. The enzyme was still active in the aroma of the pudding. In the other batch, liquefaction occurred due to a protease. In the liquefied batch of vanilla pudding, on the other hand, proteolytic ATP-dependent Clp protease of *Pseudomonas savastanoi pv. Phaseolicola* was determined as the cause of liquefaction through cleavage of caseins. With these data, we provide novel insights on the bioactive function of additive-derived components and their impact on the composition and quality of dairy products. These findings may help to prevent liquefaction of said dairy products through adjustment of processing protocols and/or additive composition.

## Data Availability

Data is contained within the article or Supplementary Materials.

## Supplementary Materials

The following data are available online at https://www.mdpi.com/article/10.3390/metabo12030254/s1. Table S1: Proteins identified from SDS gels; Table S2: Proteins identified in amylase preparations; Table S3: Proteins identified in chocolate pudding aroma; Table S4: Proteins identified in liquefied vanilla pudding.

## References

[1] Rysstad G., Kolstad J. (2006). Extended shelf life milk-advances in technology. Int. J. Dairy Technol..

[2] Schmidt V.S., Kaufmann V., Kulozik U., Scherer S., Wenning M. (2012). Microbial biodiversity, quality and shelf life of microfiltered and pasteurized extended shelf life (ESL) milk from Germany, Austria and Switzerland. Int. J. Food Microbiol..

[3] Paul J.S., Gupta N., Beliya E., Tiwari S., Jadhav S.K. (2021). Aspects and Recent Trends in Microbial alpha-Amylase: A Review. Appl Biochem. Biotechnol..

[4] Mehta D., Satyanarayana T. (2016). Bacterial and Archaeal alpha-Amylases: Diversity and Amelioration of the Desirable Characteristics for Industrial Applications. Front. Microbiol..

[5] Pandey A., Nigam P., Soccol C.R., Soccol V.T., Singh D., Mohan R. (2000). Advances in microbial amylases. Biotechnol. Appl. Biochem..

[6] de Souza P.M., de Oliveira Magalhaes P. (2010). Application of microbial alpha-amylase in industry—A review. Braz. J. Microbiol..

[7] Vincent D., Ezernieks V., Elkins A., Nguyen N., Moate P.J., Cocks B.G., Rochfort S. (2015). Milk Bottom-Up Proteomics: Method Optimization. Front. Genet..

[8] Geyer P.E., Holdt L.M., Teupser D., Mann M. (2017). Revisiting biomarker discovery by plasma proteomics. Mol. Syst. Biol..

[9] Roncada P., Piras C., Soggiu A., Turk R., Urbani A., Bonizzi L. (2012). Farm animal milk proteomics. J. Proteomics.

[10] Vandooren J., Geurts N., Martens E., Van den Steen P.E., Opdenakker G. (2013). Zymography methods for visualizing hydrolytic enzymes. Nat. Methods.

[11] Stam M.R., Danchin E.G., Rancurel C., Coutinho P.M., Henrissat B. (2006). Dividing the large glycoside hydrolase family 13 into subfamilies: Towards improved functional annotations of alpha-amylase-related proteins. Protein Eng. Des. Sel..

[12] Centeno-Leija S., Espinosa-Barrera L., Velazquez-Cruz B., Cardenas-Conejo Y., Virgen-Ortiz R., Valencia-Cruz G., Saenz R.A., Marin-Tovar Y., Gomez-Manzo S., Hernandez-Ochoa B. (2022). Mining for novel cyclomaltodextrin glucanotransferases unravels the carbohydrate metabolism pathway via cyclodextrins in Thermoanaerobacterales. Sci. Rep..

[13] Woo S.H., Kim J.S., Jeong H.M., Shin Y.J., Hong J.S., Choi H.D., Shim J.H. (2021). Development of Freeze-Thaw Stable Starch through Enzymatic Modification. Foods.

[14] Li Z.F., Zhang J.Y., Sun Q., Wang M., Gu Z.B., Du G.C., Wu J., Chen J. (2009). Mutations of Lysine 47 in cyclodextrin glycosyltransferase from Paenibacillus macerans enhance beta-cyclodextrin specificity. J. Agric. Food. Chem..

[15] Jurczak A., Jamka-Kasprzyk M., Bębenek Z., Staszczyk M., Jagielski P., Kościelniak D., Gregorczyk-Maga I., Kołodziej I., Kępisty M., Kukurba-Setkowicz M. (2020). Differences in Sweet Taste Perception and Its Association with the *Streptococcus mutans* Cariogenic Profile in Preschool Children with Caries. Nutrients.

[16] Mihaylova D., Popova A., Goranova Z., Petkova D., Doykina P., Lante A. (2021). The Perspective of Nectarine Fruit as a Sugar Substituent in Puddings Prepared with Corn and Rice Starch. Foods.

[17] Jeffery C.J. (2020). Enzymes, pseudoenzymes, and moonlighting proteins: Diversity of function in protein superfamilies. FEBS J..

[18] Jeffery C.J. (1999). Moonlighting proteins. Trends. Biochem. Sci..

[19] Jeffery C.J. (2003). Moonlighting proteins: Old proteins learning new tricks. Trends. Genet..

[20] Jeffery C.J. (2017). Moonlighting proteins—Nature’s Swiss army knives. Sci. Prog..

[21] Chen C., Zabad S., Liu H., Wang W., Jeffery C. (2018). MoonProt 2.0: An expansion and update of the moonlighting proteins database. Nucleic Acids Res..

[22] Ranjani V., Janeček S., Chai K.P., Shahir S., Abdul Rahman R.N., Chan K.G., Goh K.M. (2014). Protein engineering of selected residues from conserved sequence regions of a novel Anoxybacillus α-amylase. Sci. Rep..

[23] van der Kaaij R.M., Janeček Š., van der Maarel M., Dijkhuizen L. (2007). Phylogenetic and biochemical characterization of a novel cluster of intracellular fungal alpha-amylase enzymes. Microbiology.

[24] Janeček Š., Svensson B., MacGregor E.A. (2014). α-Amylase: An enzyme specificity found in various families of glycoside hydrolases. Cell. Mol. Life Sci..

[25] Wang Y., Li F., Zhang Y. (2010). Preliminary investigation on the action modes of an oligosaccharide-producing multifunctional amylase. Appl. Biochem. Biotechnol..

[26] Mehta D., Satyanarayana T. (2013). Dimerization mediates thermo-adaptation, substrate affinity and transglycosylation in a highly thermostable maltogenic amylase of Geobacillus thermoleovorans. PLoS ONE.

[27] Li F., Zhu X., Li Y., Cao H., Zhang Y. (2011). Functional characterization of a special thermophilic multifunctional amylase OPMA-N and its N-terminal domain. Acta. Biochim. Biophys. Sin..

[28] Tsunasawa S., Masaki T., Hirose M., Soejima M., Sakiyama F. (1989). The primary structure and structural characteristics of Achromobacter lyticus protease I, a lysine-specific serine protease. J. Biol. Chem..

[29] Slifkin M., Cumbie R. (1987). Serogrouping single colonies of beta-hemolytic streptococci with achromopeptidase extraction. J. Clin. Microbiol..

[30] Biwer A., Antranikian G., Heinzle E. (2002). Enzymatic production of cyclodextrins. Appl. Microbiol. Biotechnol..

[31] Gonzalez Pereira A., Carpena M., Garcia Oliveira P., Mejuto J.C., Prieto M.A., Simal Gandara J. (2021). Main Applications of Cyclodextrins in the Food Industry as the Compounds of Choice to Form Host-Guest Complexes. Int. J. Mol. Sci..

[32] Lim C.H., Rasti B., Sulistyo J., Hamid M.A. (2021). Comprehensive study on transglycosylation of CGTase from various sources. Heliyon.

[33] Lin Y.K., Show P.L., Yap Y.J., Ariff A.B., Mohammad Annuar M.S., Lai O.M., Tang T.K., Juan J.C., Ling T.C. (2016). Production of gamma-cyclodextrin by *Bacillus cereus* cyclodextrin glycosyltransferase using extractive bioconversion in polymer-salt aqueous two-phase system. J. Biosci. Bioeng..

[34] Leemhuis H., Kelly R.M., Dijkhuizen L. (2010). Engineering of cyclodextrin glucanotransferases and the impact for biotechnological applications. Appl. Microbiol. Biotechnol..

[35] Saini K., Kashyap A., Saini M., Gupta R. (2022). Gamma cyclodextrin glycosyltransferase from evansella caseinilytica: Production, characterization and product specificity. 3 Biotech.

[36] Jemli S., Jaoua M., Bejar S. (2016). US132 Cyclodextrin Glucanotransferase Engineering by Random Mutagenesis for an Anti-Staling Purpose. Mol. Biotechnol..

[37] Sonnendecker C., Zimmermann W. (2019). Domain shuffling of cyclodextrin glucanotransferases for tailored product specificity and thermal stability. FEBS Open Bio.

[38] Ottoni J.R., e Silva T.R., de Oliveira V.M., Passarini M.R.Z. (2020). Characterization of amylase produced by cold-adapted bacteria from Antarctic samples. Biocatal. Agric. Biotechnol..

[39] Couto S.R., Sanromán M.Á. (2006). Application of solid-state fermentation to food industry—A review. J. Food Eng..

[40] Tamman H., Ainelo A., Tagel M., Hõrak R. (2015). Stability of the GraA Antitoxin Depends on Growth Phase, ATP Level, and Global Regulator MexT. J. Bacteriol..

[41] Hall B.M., Breidenstein E.B.M., de la Fuente-Núñez C., Reffuveille F., Mawla G.D., Hancock R.E.W., Baker T.A. (2017). Two Isoforms of Clp Peptidase in Pseudomonas aeruginosa Control Distinct Aspects of Cellular Physiology. J. Bacteriol..

[42] Jin Y., Yu Y., Qi Y., Wang F., Yan J., Zou H. (2016). Peptide profiling and the bioactivity character of yogurt in the simulated gastrointestinal digestion. J. Proteomics.

[43] Bradford M.M. (1976). A rapid and sensitive method for the quantitation of microgram quantities of protein utilizing the principle of protein-dye binding. Anal. Biochem..

[44] Grosche A., Hauser A., Lepper M.F., Mayo R., von Toerne C., Merl-Pham J., Hauck S.M. (2016). The Proteome of Native Adult Muller Glial Cells from Murine Retina. Mol. Cell Proteomics.

[45] Wisniewski J.R., Zielinska D.F., Mann M. (2011). Comparison of ultrafiltration units for proteomic and N-glycoproteomic analysis by the filter-aided sample preparation method. Anal. Biochem..

[46] Kleinwort K.J.H., Degroote R.L., Hirmer S., Korbonits L., Lorenz L., Scholz A.M., Hauck S.M., Deeg C.A. (2022). Bovine Peripheral Blood Derived Lymphocyte Proteome and Secretome Show Divergent Reaction of Bovine Immune Phenotypes after Stimulation with Pokeweed Mitogen. Proteomes.

